# Understanding the Prevalence and Risk Factor Profile of Olfactory Impairment and Its Impact on Patient Health Indicators and Economic Outcomes in Community-Dwelling Older Asian Adults

**DOI:** 10.1093/geroni/igae088

**Published:** 2024-09-27

**Authors:** Ryan E K Man, Chiew Meng Johnny Wong, Preeti Gupta, Eva K Fenwick, Amudha Aravindhan, Neville Wei Yang Teo, Tze Choong Charn, Ciaran Forde, Ecosse L Lamoureux

**Affiliations:** Singapore Eye Research Institute, Singapore National Eye Centre, Singapore, Singapore; Duke-NUS Medical School, Singapore, Singapore; Singapore Eye Research Institute, Singapore National Eye Centre, Singapore, Singapore; Singapore Eye Research Institute, Singapore National Eye Centre, Singapore, Singapore; Duke-NUS Medical School, Singapore, Singapore; Singapore Eye Research Institute, Singapore National Eye Centre, Singapore, Singapore; Duke-NUS Medical School, Singapore, Singapore; Singapore Eye Research Institute, Singapore National Eye Centre, Singapore, Singapore; Duke-NUS Medical School, Singapore, Singapore; Duke-NUS Medical School, Singapore, Singapore; Department of Otorhinolaryngology, Singapore General Hospital, Singapore, Singapore; Department of Otorhinolaryngology, Singapore General Hospital, Singapore, Singapore; Division of Human Nutrition and Health, Wageningen University and Research, The Netherlands; Singapore Eye Research Institute, Singapore National Eye Centre, Singapore, Singapore; Duke-NUS Medical School, Singapore, Singapore

**Keywords:** Anosmia, Economic burden, Hyposmia, Olfaction, Risk factors

## Abstract

**Background and Objectives:**

There is a paucity of data on the prevalence, risk factors, and impact of olfactory impairment (OI) on key health indicators and economic outcomes in Asian populations. We aimed to address these gaps in a population of community-dwelling older adults.

**Research Design and Methods:**

We included 2 101 participants (mean age ± standard deviation [*SD*]: 72.9 ± 8.1 years; 55.1% women) from the baseline assessment of the Population Health and Eye Disease Profile in Elderly Singaporeans (PIONEER) study (2017–2022). Any OI was based on a score of <11 on the 16-item identification segment of the Sniffin’ Sticks test battery; subcategorized into hyposmia (score 9–10) and anosmia (score ≤8). Sociodemographic, clinical, and lifestyle risk determinants, health indicators (health-related quality of life, depressive symptoms, daily caloric intake, frailty, and cognitive impairment), and economic outcomes (healthcare expenditure, productivity loss) were assessed via standardized clinical testing and validated questionnaires. Multivariable logistic and linear regression models were utilized to explore the risk factor profile of OI across its severity spectrum and its impact on health indicators and economic outcomes.

**Results:**

The census-adjusted prevalence of any OI, hyposmia, and anosmia were 34.0%, 20.5%, and 13.5%, respectively. Older age and male gender were associated with increased likelihood of hyposmia and anosmia, while the presence of diabetes and >4 days/week alcohol consumption were associated with increased odds of having anosmia only (all *p* < .05). Both hyposmia and anosmia were also associated with more than twofold increased odds of having CI.

**Discussion and Implications:**

Over a third of our community-dwelling older Singaporean population had OI, with 1-in-10 experiencing total olfaction loss. Those with OI had more than double the odds of having CI, regardless of its severity. Our results suggest the importance of community-based programs aimed at detecting and delaying the progression of OI in high-risk individuals.

**Translational Significance:** This study explored the prevalence, risk factor profile, and impact of olfactory impairment (OI) on key patient health indicators and economic outcomes in community-dwelling older Asian adults. We found over one third of older Asian adults had OI, with 1-in-10 experiencing total loss of smell, assessed objectively using the Sniffin’ Sticks test. Increasing age, male gender, regular alcohol consumption, and diabetes were key drivers; with OI found to be associated with a higher likelihood of cognitive impairment. Our findings advocate for targeted implementation of OI screening and intervention programs to delay the progression of CI in high-risk individuals.

Olfaction, that is, the ability to detect scents in the environment, is one of the 5 key pathways by which the body receives sensory input from the environment (1). Although olfactory impairment (OI) has historically received less attention than other sensory systems, such as vision and hearing, the widespread occurrence of anosmia (loss of smell) as a hallmark symptom of coronavirus disease 2019 [COVID-19] (2), has recently brought OI into the spotlight, prompting interest in its prevalence, contributing factors, and impact on affected individuals.

The presence of OI is typically assessed clinically using detailed psychological testing, for example, the Sniffin’ Sticks test battery, comprising odor identification, discrimination, and threshold tests (3,4), or electrophysiologically using electro-olfactograms (EOGs) or odor event-related potentials (OERPs) (5). Due to the need for specialized equipment and the length of these tests, primary screening and epidemiological prevalence studies often utilize screening tests (6), such as the odor identification portion of the Sniffin’ Sticks test (Sniffin’ Sticks Identification Test [SSIT]) or self-reported questionnaires like the Questionnaire of Olfactory Disorders (7).

Epidemiological studies using these screening tests have reported OI prevalence rates ranging between 2.63% and 67.7%, depending on the study population and method of olfaction assessment (8,9), with common risk factors being older age; male gender; African American ethnicity; smoking; history of head trauma and solvent exposure; and heavy alcohol intake (10,11). However, most of these studies were carried out in Western populations, with comparatively limited information available from Asian regions. For instance, only 5 out of 25 studies included in a recent meta-analysis were conducted in Asian populations (8). In addition, 3 of these 5 studies used self-reporting to determine the presence of OI, which may lead to underestimation of true OI rates due to perception bias (8,12). Moreover, while there is increasing evidence that OI is associated with the worsening of key clinical and patient-centered health outcomes that are themselves associated with increased morbidity and mortality, such as a greater risk of neurodegenerative diseases (13); poorer mental well-being including increased likelihood of depression (14) and poorer quality of life (QoL) (15); altered appetite and metabolism (16,17); and greater likelihood of frailty (18). However, these findings were derived from a largely Caucasian sample and may not be fully generalizable to Asians due to several factors including differences in genetic predisposition (19); socioeconomic disparities that may impact odor identification (20); differences in prevalence rates of medical conditions that can impact olfaction (21); as well as differences in lifestyle factors (eg, smoking), cultural beliefs and health-seeking behavior (22,23).

To address these knowledge gaps, we determined the prevalence, associated risk determinants, and impact of OI on the above health indicators (daily caloric intake, health-related QoL (HRQoL), depressive symptoms, frailty, and cognitive impairment [CI]) and economic outcomes (direct healthcare expenditure, productivity loss) in a population-based study of older adults aged ≥60 years using the SSIT. We hypothesize that the rates of OI will be higher than that reported in the recent meta-analysis of Asian studies by virtue of our objective OI evaluation methodology that is likely to be more accurate than the self-reported measures used in the majority of current studies; that our risk determinants across the OI severity spectrum may differ from that found in Western populations; and that those with OI will have significantly worse impact on key health indicators and economic outcomes compared with those without OI.

## Method

### Study Population

The Population Health and Eye Disease Profile in Elderly Singaporeans (PIONEER) is a population-based cohort study that aims to evaluate the epidemiology, risk factors, and patient-centered and economic impact of age-related sensory loss, together with its overarching relationship with systemic aging. The baseline visit was conducted between 2017 and 2022 among older Chinese, Malay, and Indian adults living in Singapore (24). A detailed sampling strategy can be found in the Supplementary Materials; in brief, participants were included if they were Singaporean nationals or permanent residents aged ≥60 years, were community-dwelling individuals (ie, not residing in nursing homes or institutionalized facilities), and were able to give informed consent (ie, no severe cognitive, hearing or speech impairments). The study protocol followed the declaration of Helsinki and ethics approval from Singapore’s Centralized Institutional Review Board was obtained before the study began recruitment (No. 2016/3089).

### Assessment of Olfactory Impairment

The olfactory function of participants was assessed using the 16-item SSIT (25). Participants were asked to identify 16 different odors (eg, coffee, shoe leather, orange) from felt pen tips impregnated with 4 mL of the odorant at intervals of 30 seconds between each odor presentation. Participants were then tasked to choose the correct answer from 4 multiple-choice options. Any OI was defined as an SSIT score of <11 based on updated cut points published by Hummel and colleagues, and further categorized into hyposmia (decreased sense of smell; SSIT score 9–10), and anosmia (SSIT score ≤8) (3). As these cut points were derived from Caucasian individuals, we conducted sensitivity analyses with our own cut points generated by following the protocol pioneered by Hummel’s group. Odors with ≤50% correct identification rates by the overall sample were excluded (apple and turpentine; Supplementary Table 1), after which cut points for any hyposmia were generated based on the 10th percentile test score across all subjects (SSIT < 7) (25). However, it was not possible to carry out corresponding analyses of anosmia using this method as Hummel’s group recruited clinically diagnosed anosmic patients rather than utilize percentile-based analyses to derive cut points for anosmia (25).

### Assessment and Definition of Covariables

Participants’ sociodemographic details, including age, gender, ethnicity, socioeconomic status (comprising income and education), self-reported medical history [presence of diabetes, hypertension, asthma, dyslipidemia, cardiovascular disease (CVD), including ischemic heart disease and stroke; chronic kidney disease (CKD), neurological disorders (Parkinson’s, multiple sclerosis, migraines not inclusive of mild CI or dementia), neuropsychiatric disorders (schizophrenia, depression)], and lifestyle factors (smoking status, frequency of alcohol consumption and weekly duration spent on moderate–vigorous physical activity levels, eg, gardening, brisk walking, dancing, jogging) were collected via an in-house questionnaire. Low socioeconomic status was operationally defined as having primary or lower education and individual monthly income <SGD2000, and low moderate–vigorous levels of physical activity were defined as the gender-specific lowest quintile of total self-reported duration of moderate–vigorous physical activity levels. Systolic and diastolic blood pressures were measured twice using an automated blood pressure (Dinamap Pro Series DP110X-RW; GE Medical Systems Information Technologies, Inc), with a third reading taken if the 2 previous systolic or diastolic readings differed by more than 10 or 5 mm Hg, respectively. The mean of the closest 2 readings was used in analyses. Blood samples were collected for hemoglobin A1c (HbA1c); random glucose; total, high-density lipoprotein, and low-density lipoprotein cholesterol; and triglyceride levels. Finally, participants’ total fat mass was derived from a whole-body dual-energy x-ray absorptiometry scan (Hologic Discovery W; Hologic Inc, Marlborough, MA).

Hypertension was defined as SBP ≥ 140 mm Hg, DBP ≥ 90 mm Hg, self-reported use of antihypertensive medications, or self-reported history of physician-diagnosed hypertension. Diabetes was defined as random glucose ≥11.1 mmol/L, HbA1c ≥ 6.5%, self-reported use of antidiabetic medication, or reported history of physician-diagnosed diabetes. Dyslipidemia was defined as total cholesterol ≥5.2 mmol/L or low-density-lipoprotein cholesterol ≥3.4 mmol/L or triglycerides ≥1.7 mmol/L or self-reported use of lipid-lowering medications. Fat mass index (FMI) was calculated as fat mass/height (2,24) with high FMI defined as an FMI score >8.51 and 11.6 for men and women, respectively (26). CVD was defined as a self-reported history of myocardial infarction/angina and stroke. Neuropsychiatric disorder was considered present via patient self-report and if the participant reported taking antidepressants of any kind (e.g., Cymbalta or any selective serotonin reuptake inhibitors). CKD was defined as an estimated glomerular filtration rate < 60 mL/min/1.73 m^2^ and via patient self-reported history.

### Assessment of Health Indicators and Economic Outcomes

HRQoL was assessed using the EuroQoL 5-Dimension (EQ-5D-5L) questionnaire, a preference-based measure measuring QoL in 5 dimensions (Mobility, Self-care, Usual Activities, Pain/Discomfort, and Anxiety/Depression) (27). A utility index, ranging from 1 (perfect health) to −0.594 (worse than death) was calculated for each individual using established UK EQ-5D tariffs (28). The 9-item patient health questionnaire was used to screen for depressive symptoms, with higher scores indicating worse symptoms (29). Dietary (nutrition) data were collected using an electronic Food Frequency Questionnaire developed and validated for the local multiethnic Singaporean population (30). These data were then converted to caloric intake/day in kilocalories (kcal). Participants’ height and weight were measured using a wall-mounted, adjustable measuring scale and a calibrated scientific weight scale, respectively; with body mass index calculated as weight (kg)/height (m) (2). Gait speed was assessed using a 4 m gait speed test, and grip strength was measured with a digital hand dynamometer (JAMAR Plus+, JLW instruments, Chicago, IL). Frailty was defined using the modified Fried Frailty criteria based on the presence of any 3 or more of the following (31): unintentional shrinkage, defined as body mass index <18.5 kg/m^2^; slowness, defined as the gender-specific lowest quintile of gait speed; weakness, defined as the gender-specific lowest quintile of grip strength; exhaustion, defined as a total score of <10 for 3 questions from the vitality domain of the 12-item short-form survey assessed as part of our in-house questionnaire administration; and low moderate–vigorous physical activity levels, as defined in the previous section. Lastly, the participant’s cognitive ability was assessed using both the Montreal Cognitive Assessment test—Basic (MoCA-Basic), with CI defined as a score of <19, <22, and <24 for individuals with ≤6, 7–12, and >12 years of education, respectively (32), as well as the 6-item CI test, with CI defined as a score ≥8 (33).

The modified healthcare services expenditure module (34) was used to assess direct healthcare expenditure, comprising hospitalization and emergency department visit cost over the past 6 months, and mental health and outpatient service utilization over the past 3 months, extrapolated to annual healthcare expenditure. Productivity loss over the last 7 days for working individuals, extrapolated to the participant’s annual income, was also captured with this module.

### Statistical Analysis

All statistical evaluations were conducted using R software version 3.4.1 (R Core Team, 2023) and assumed a 2-sided test at the 5% significance level. Participant sociodemographic, medical, clinical, and lifestyle characteristics were summarized using means (standard deviation [*SD*]) for continuous variables and *N* (%) for categorical variables. As we oversampled minority races, women, and older participants, the overall, age-, gender-, and ethnicity-stratified prevalence rates for any OI, hyposmia, and anosmia were determined by weighing individuals according to their sampling probabilities and standardizing to Singapore’s 2020 population census.

To determine the sociodemographic, medical, clinical, and lifestyle factors associated with hyposmia and anosmia, we utilized multinomial logistic regression models. Variables included in the model were age, gender, ethnicity, low socioeconomic status, high FMI, smoking status, frequency of alcohol consumption, low moderate–vigorous physical activity levels, and the presence of systemic comorbidities (diabetes, hypertension, dyslipidemia, CVD, CKD, asthma, neurological, and neuropsychiatric disorders).

Lastly, to evaluate the associations between OI across its severity spectrum with key health indicators and economic outcomes, we utilized generalized linear models for continuous health indicators (HRQoL, depressive symptoms, and daily caloric intake) and logistic regression models for binary outcomes (frailty and CI). For economic outcomes (ie, direct healthcare expenditure and productivity loss), we employed 2-part models combining both logistic and gamma generalized linear models with a log-link function to estimate the total marginal effect of hyposmia and anosmia on these outcomes. Furthermore, all models were adjusted for confounders (age, gender, ethnicity) and variables that were identified as potential risk factors for OI in the literature.

## Results

Of the 2,643 participants recruited in the PIONEER baseline assessment, 7 (0.3%) were excluded because they failed to meet the age and ethnicity inclusion criteria, and a further 535 (20.2%) were excluded as they did not have SSIT data. These exclusions left 2 101 (79.5%) individuals (mean age ± *SD*: 72.9 ± 8.1 years; 55.1% women) for analyses. Of these, 1,010 (48.1%), 605 (28.8%), and 486 (23.1%) were of Chinese, Malay, and Indian ethnicities, respectively (Table 1).

**Table 1.** Demographic, Systemic, and Socioeconomic Characteristics of Participants Stratified by OI Status (Hyposmia, Anosmia)

Characteristics	No OI	Hyposmia	Anosmia	Overall
	(*n* = 1260)	(*n* = 483)	(*n* = 358)	(*N* = 2101)
	Mean (*SD*) or *n* (%)			
Age (years)	70.7 (7.6)	75.1 (7.9)	77.3 (7.6)	72.9 (8.1)
Age group				
60–69	631 (50.1)	127 (26.3)	66 (18.4)	824 (39.2)
70–79	410 (32.5)	185 (38.3)	117 (32.7)	712 (33.9)
≥80	219 (17.4)	171 (35.4)	175 (48.9)	565 (26.9)
Women	751 (59.6)	253 (52.4)	153 (42.7)	1157 (55.1)
Ethnicity				
Chinese	612 (48.6)	227 (47.0)	171 (47.8)	1010 (48.1)
Malay	362 (28.7)	133 (27.5)	110 (30.7)	605 (28.8)
Indian	286 (22.7)	123 (25.5)	77 (21.5)	486 (23.1)
High FMI	516 (41.0)	183 (37.9)	130 (36.3)	829 (39.5)
Occupation				
No occupation/unemployed	40 (3.2)	11 (2.3)	8 (2.2)	59 (2.8)
Employed	354 (28.1)	109 (22.6)	66 (18.4)	529 (25.2)
Homemakers	146 (11.6)	42 (8.7)	39 (10.9)	227 (10.8)
Retired	677 (53.7)	298 (61.7)	223 (62.3)	1198 (57.0)
Low socioeconomic status	200 (15.9)	84 (17.4)	72 (20.1)	356 (16.9)
Smoking status				
Never smoked	960 (76.2)	334 (69.2)	239 (66.8)	1533 (73.0)
Past smoker	171 (13.6)	82 (17.0)	77 (21.5)	330 (15.7)
Current smoker	101 (8.0)	52 (10.8)	25 (7.0)	178 (8.5)
Alcohol consumption				
None	1077 (85.5)	414 (85.7)	292 (81.6)	1783 (84.9)
≤4 days per week	90 (7.1)	31 (6.4)	27 (7.5)	148 (7.0)
>4 days per week	22 (1.7)	11 (2.3)	13 (3.6)	46 (2.2)
Low MVPA level	743 (59.0)	258 (53.4)	217 (60.6)	1218 (58.0)
Systemic conditions				
Cardiovascular disease	203 (16.1)	79 (16.4)	76 (21.2)	358 (17.0)
Diabetes	399 (31.7)	167 (34.6)	145 (40.5)	711 (33.8)
Chronic kidney disease	190 (15.1)	104 (21.5)	99 (27.7)	393 (18.7)
Asthma	113 (9.0)	41 (8.5)	27 (7.5)	181 (8.6)
Hypertension	1057 (83.9)	422 (87.4)	311 (86.9)	1790 (85.2)
Dyslipidaemia	1053 (83.6)	393 (81.4)	279 (77.9)	1725 (82.1)
Neurological disorders	151 (12.0)	41 (8.5)	18 (5.0)	210 (10.0)
Neuropsychiatric disorders	52 (4.1)	27 (5.6)	19 (5.3)	98 (4.7)

*Notes*: BP = blood pressure; LDL = low-density lipoprotein; MVPA = moderate–vigorous physical activity; OI = olfactory impairment; Q1 = 25th percentile; Q3 = 75th percentile; *SD* = standard deviation. High FMI = High fat mass index (FMI) defined as FMI > 8.51 for men and FMI > 11.6 for women; Low Socioeconomic Status: Having primary or lower education and individual monthly income < SGD2000; Low MVPA level: Gender-specific lowest quintile of total self-reported duration spent carrying out moderate and vigorous activity (e.g., gardening, brisk walking, dancing, jogging); cardiovascular disease: Self-reported history of angina, heart attack, heart disease, or stroke; Diabetes: HbA1c > 6.5%, random blood glucose ≥ 11.1 mmol/L, use of diabetic medication and self-reported; Chronic Kidney Disease: Estimated glomerular filtration rate < 60 mL/min/1.73 m^2^; asthma: self-reported; hypertension: systolic BP ≥ 140 mm Hg, diastolic BP ≥ 90 mm Hg, physician diagnosis, use of BP medication and/or self-report; dyslipidemia: total cholesterol ≥ 5.2 mmol/L or LDL cholesterol ≥ 3.4 mmol/L or triglycerides ≥ 1.7 mmol/L or use of anti-cholesterol medication; neurological disorders: self-reported history of neurological disorders (e.g., Parkinsons, multiple sclerosis, migraines); neuropsychiatric disorders: self-reported history of neuropsychiatric (including depression) disorders or any use of duloxetine (Cymbalta) or the class of selective serotonin reuptake inhibitors (SSRIs) medications.

OI was present in 841 of the 2 101 (40.0%) individuals analyzed, comprising 483 (23.0%) with hyposmia, and 358 (17.0%) with anosmia. The census-weighted prevalence rates were 34.0% (95% CI: 31.4%–36.6%) for any OI, 20.5% (95% CI: 18.3%–22.8%) for hyposmia, and 13.5% (95% CI: 11.7%–15.4%) for anosmia (Table 2). OI prevalence rates rose with age, from 24.5% at ages 60–69 years to 58.0% for those ≥80 years (*p* trend <.001), with similar trends observed for the prevalence of hyposmia and anosmia (Table 2). The prevalence rates of OI across its severity spectrum were also markedly higher in men compared with women, although there were no notable differences across the 3 ethnicities (Table 2). In sensitivity analyses utilizing the population-specific cut point for hyposmia (SSIT < 7), we found a census-weighted OI prevalence of 5.9% (95% CI: 4.8%–7.2%; Supplementary Table 2).

**Table 2.** Prevalence of OI Across Its Severity Spectrum, Stratified by Age, Gender, and Ethnicity in the PIONEER Study

Variable	All (*N* = 2101)		Gender				Ethnicity					
			Men (*n* = 944)		Women (*n* = 1157)		Chinese (*n* = 1010)		Malay (*n* = 605)		Indian (*n* = 486)	
	*n*	Weighted, % (95% CI)	*n*	Weighted, % (95% CI)	*n*	Weighted, % (95% CI)	*n*	Weighted, % (95% CI)	*n*	Weighted, % (95% CI)	*n*	Weighted, % (95% CI)
Any OI												
60–69	193	24.5 (20.9–28.4)	124	32.5 (26.8–38.6)	69	16.7 (12.3–21.8)	90	24.8 (20.5–29.6)	54	21.0 (16.3–26.4)	49	26.0 (19.9–32.8)
70–79	302	41.2 (36.8–45.7)	156	48.3 (41.2–55.5)	146	35.0 (29.5–40.8)	147	40.5 (35.4–45.7)	91	47.9 (40.6–55.3)	64	42.1 (34.2–50.3)
≥80	346	58.0 (52.2–63.6)	155	59.0 (50.8–66.8)	191	57.4 (49.4–65.1)	161	56.7 (50.1–63.1)	98	69.6 (61.1–77.2)	87	63.1 (54.3–71.4)
* p*-trend	<0.001		<0.001		<0.001		<0.001		<0.001		<0.001	
Total	841	34.0 (31.4–36.6)	435	40.1 (36.0–44.3)	406	28.6 (25.4–31.9)	398	34.1 (31.1–37.2)	243	32.7 (29.0–36.5)	200	34.6 (30.2–39.3)
Hyposmia												
60–69	127	16.2 (13.1–19.6)	77	21.4 (16.5–27.0)	50	11.1 (7.6–15.6)	60	16.6 (12.9–20.8)	33	12.6 (8.9–17.3)	34	17.8 (12.7–24.1)
70–79	185	24.8 (21.0–28.9)	88	27.0 (21.0–33.7)	97	22.9 (18.2–28.2)	89	24.3 (19.9–29.0)	54	28.5 (22.1–35.5)	42	27.3 (20.4–35.1)
≥80	171	29.1 (24.1–34.6)	65	27.7 (20.8–35.6)	106	30.0 (23.1–37.6)	78	28.6 (22.9–34.8)	46	32.0 (23.8–41.1)	47	33.5 (25.8–42.0)
* p*-trend	<0.001		0.051		<0.001		<0.001		<0.001		<0.001	
Total	483	20.5 (18.3–22.8)	230	23.7 (20.2–27.5)	253	17.6 (14.9–20.6)	227	20.6 (18.0–23.4)	133	18.5 (15.4–22.0)	123	22.2 (18.3–26.5)
Anosmia												
60–69	66	8.3 (6.1–11.0)	47	11.1 (7.6–15.5)	19	5.5 (3.0–9.2)	30	8.3 (5.7–11.6)	21	8.4 (5.3–12.5)	15	8.2 (4.7–13.0)
70–79	117	16.4 (13.2–20.0)	68	21.4 (15.9–27.7)	49	12.1 (8.6–16.4)	58	16.2 (12.5–20.4)	37	19.5 (14.1–25.9)	22	14.8 (9.6–21.5)
≥80	175	28.9 (24.0–34.1)	90	31.3 (24.4–38.8)	85	27.4 (20.9–34.8)	83	28.1 (22.6–34.1)	52	37.6 (29.0–46.8)	40	29.6 (22.0–38.2)
* p*-trend	<0.001		<0.001		<0.001		<0.001		<0.001		<0.001	
Total	358	13.5 (11.7–15.4)	205	16.4 (13.5–19.5)	153	11.0 (8.9–13.4)	171	13.5 (11.4–15.8)	110	14.1 (11.5–17.2)	77	12.5 (9.6–15.8)

*Notes*: CI = confidence interval; OI = olfactory impairment. Weighted prevalences were calculated with sampling weights specific to each age group, gender and ethnicity to adjust for oversampling and post-stratification weights to align to the population distribution based on the 2020 Singapore Census.

In multivariable models evaluating the risk determinants of OI across the severity spectrum, we found that older age and male gender were associated with an increased likelihood of having hyposmia [odds ratio (OR): 1.10, 95% confidence interval (CI): 1.07–1.13, *p* < .001 per year increase in age; and OR: 1.66, 95% CI: 1.08–2.55, *p* = .020 for male gender] and anosmia (OR: 1.14, 95% CI: 1.11–1.18, *p* < .001 per year increase in age; and OR: 2.26, 95% CI: 1.34–3.80, *p* = .002 for male gender; Table 3). Additionally, an alcohol consumption frequency of >4 days/week (OR: 3.04, 95% CI: 1.17–7.86, *p* = .022), and the presence of diabetes (OR: 1.59, 95% CI: 1.01–2.50, *p* = .045) were both associated with increased odds of anosmia. Conversely, both low levels of moderate–vigorous physical activity (OR: 0.61, 95% CI: 0.43–0.87, *p* = .006) and the presence of CKD (OR: 0.60, 95% CI: 0.37–0.97, *p* = .037) were associated with a reduced likelihood of hyposmia (Table 3). In sensitivity analyses, older age, and Malay ethnicity were all associated with an increased likelihood of any OI, while having a high FMI was associated with lower odds of having any OI (Supplementary Table 3).

**Table 3.** Risk Determinants of OI Severity (Hyposmia and Anosmia)

Variable	Comparison of OI status			
	Hyposmia vs no OI		Anosmia vs no OI	
	OR (95% CI)	*p* value	OR (95% CI)	*p* Value
Age (years)	1.10 (1.07–1.13)	<0.001	1.14 (1.11–1.18)	<0.001
Gender				
Women	Reference	NA	Reference	NA
Men	1.66 (1.08–2.55)	0.020	2.26 (1.34–3.80)	0.002
Ethnicity				
Chinese	Reference	NA	Reference	NA
Malay	0.88 (0.56–1.38)	0.576	1.33 (0.76–2.31)	0.316
Indian	1.09 (0.68–1.75)	0.711	0.75 (0.40–1.41)	0.370
High FMI				
No	Reference	NA	Reference	NA
Yes	0.84 (0.58–1.22)	0.358	0.73 (0.46–1.17)	0.192
Low socioeconomic status				
No	Reference	NA	Reference	NA
Yes	0.91 (0.57–1.46)	0.697	0.76 (0.42–1.37)	0.366
Smoking status				
Never smoked	Reference	NA	Reference	NA
Past smoker	1.16 (0.70–1.91)	0.569	0.98 (0.55–1.76)	0.948
Current smoker	1.74 (0.94–3.21)	0.076	0.71 (0.30–1.67)	0.434
Alcohol consumption				
None	Reference	NA	Reference	NA
≤4 days/week	0.81 (0.45–1.43)	0.459	1.07 (0.52–2.20)	0.851
>4 days/week	0.75 (0.26–2.21)	0.603	3.04 (1.17–7.86)	0.022
Low MVPA level				
No	Reference	NA	Reference	NA
Yes	0.61 (0.43–0.87)	0.006	0.73 (0.47–1.14)	0.171
Cardiovascular disease				
No	Reference	NA	Reference	NA
Yes	1.00 (0.63–1.58)	0.987	1.14 (0.66–1.96)	0.636
Diabetes				
No	Reference	NA	Reference	NA
Yes	1.24 (0.86–1.79)	0.255	1.59 (1.01–2.50)	0.045
Chronic kidney disease				
No	Reference	NA	Reference	NA
Yes	0.60 (0.37–0.97)	0.037	0.96 (0.57–1.62)	0.879
Asthma				
No	Reference	NA	Reference	NA
Yes	1.26 (0.72–2.22)	0.419	0.96 (0.45–2.06)	0.925
Hypertension				
No	Reference	NA	Reference	NA
Yes	1.27 (0.74–2.16)	0.381	0.73 (0.38–1.40)	0.343
Dyslipidemia				
No	Reference	NA	Reference	NA
Yes	0.96 (0.54–1.71)	0.894	1.63 (0.69–3.88)	0.268
Neurological disorders				
No	Reference	NA	Reference	NA
Yes	0.78 (0.46–1.35)	0.378	0.60 (0.28–1.29)	0.193
Neuropsychiatric disorders				
No	Reference	NA	Reference	NA
Yes	1.34 (0.64–2.81)	0.44	1.60 (0.63–4.05)	0.321

*Notes*: BP = blood pressure; CI = confidence interval; LDL = low-density lipoprotein; MVPA = moderate–vigorous physical activity; NA = not applicable; OI = olfactory impairment; OR = odds ratio. High FMI = high fat mass index (FMI) defined as FMI > 8.51 for men and FMI > 11.6 for women; low socioeconomic status: having primary or lower education and individual monthly income < SGD2000; low MVPA level: Gender-specific lowest quintile of total self-reported duration spent carrying out moderate and vigorous activity (e.g., gardening, brisk walking, dancing, jogging); cardiovascular disease: self-reported history of angina, heart attack, heart disease or stroke; diabetes: HbA1c > 6.5%, random blood glucose ≥ 11.1 mmol/L, use of diabetic medication and self-reported; chronic kidney disease: estimated glomerular filtration rate < 60 mL/min/1.73 m^2^; asthma: self-reported; hypertension: systolic BP ≥ 140 mm Hg, diastolic BP ≥ 90 mm Hg, physician diagnosis, use of BP medication and self-report; dyslipidemia: total cholesterol ≥ 5.2 mmol/L or LDL cholesterol ≥ 3.4 mmol/L or triglycerides ≥ 1.7 mmol/L or use of anti-cholesterol medication; neurological disorders: Self-reported history of neurological disorders (e.g., Parkinsons, multiple sclerosis, migraines); neuropsychiatric disorders: Self-reported history of neuropsychiatric (including depression) disorders or any use of duloxetine (Cymbalta) or the class of selective serotonin reuptake inhibitors (SSRIs) medications.

Multivariable models evaluating the association between OI severity and health indicator variables revealed that both hyposmia and anosmia were associated with increased odds of having CI (OR: 2.12, 95% CI: 1.28–3.50, *p* = .003 for hyposmia; OR: 2.75, 95% CI: 1.63–4.66, *p* < .001 for anosmia; Table 4). No significant associations were found with any of the other health indicators or economic outcomes (Table 5). Sensitivity analyses revealed similar findings, with OI being associated with the presence of CI only (Supplementary Tables 4 and 5).

**Table 4.** Multivariable Associations Between OI (Hyposmia, Anosmia) and Key Health Indicators

Health Indicators	Exposure	Estimate^a^ (95% CI)	*p* Value	Overall marginal effect (95% CI)	% change
EQ-5D (HRQoL)					
	No OI	Reference	NA		
	Hyposmia	0.003 (−0.011 to 0.017)	0.701	0.003 (−0.011 to 0.017)	0.30
	Anosmia	−0.014 (−0.031 to 0.003)	0.116	−0.014 (−0.031 to 0.003)	−1.54
PHQ-9 score (depressive symptoms)					
	No OI	Reference	NA		
	Hyposmia	−0.003 (−0.228 to 0.221)	0.977	−0.003 (−0.228 to 0.221)	−0.39
	Anosmia	0.076 (−0.202 to 0.353)	0.593	0.076 (−0.201 to 0.353)	9.06
Daily caloric intake (kcal/day)					
	No OI	Reference	NA		
	Hyposmia	12.81 (−71.64 to 97.26)	0.766	12.81 (−71.55 to 97.17)	0.68
	Anosmia	25.19 (−78.12 to 128.51)	0.632	25.19 (−78.01 to 128.40)	1.35
Frailty					
	No OI	Reference	NA		
	Hyposmia	OR: 1.00 (0.66 to 1.51)	0.995	0.000 (−0.042 to 0.042)	0.12
	Anosmia	OR: 1.22 (0.76 to 1.94)	0.402	0.021 (−0.030 to 0.073)	17.78
Cognitive impairment					
	No OI	Reference	NA		
	Hyposmia	OR: 2.12 (1.28 to 3.50)	0.003	0.050 (0.015 to 0.086)	101.08
	Anosmia	OR: 2.75 (1.63 to 4.66)	<0.001	0.074 (0.031 to 0.117)	154.50

*Notes*: CI = confidence interval; EQ-5D = EuroQoL 5-dimension; HRQoL = health-related quality of life; NA =  not applicable; OI = olfactory impairment; OR = odds ratio; PHQ-9 = Patient Health Questionnaire-9; PRO = patient-reported outcome. All models are adjusted for age, gender, ethnicity, body mass index, low socioeconomic status, smoking status, cardiovascular disease, diabetes, chronic kidney disease, asthma, hypertension, and dyslipidemia.

^a^For EQ-5D, PHQ-9 and daily caloric intake, the estimates are coefficients derived from linear regression models. Frailty and cognitive impairment are OR derived from logistic regression models.

**Table 5.** Associations Between OI and Economic Outcomes Using 2-Parts Model

Cost outcome	Exposure	First part		Second part		Overall marginal effect (95% CI)
		OR (95% CI)	*p* Value	Cost ratio(95% CI)	*p* Value	
Healthcare cost	No OI	Reference	NA	Reference	NA	
	Hyposmia	0.86 (0.64 to 1.15)	0.309	1.77 (1.03 to 3.15)	0.03	195.99 (−63.51 to 455.49)
	Anosmia	0.57 (0.39 to 0.83)	0.004	0.89 (0.43 to 2.03)	0.757	−123.44 (−289.76 to 42.88)
Productivity cost^a^	No OI	Reference	NA	Reference	NA	
	Hyposmia	0.73 (0.42 to 1.29)	0.283	1.01 (0.77 to 1.33)	0.964	−491.19 (−2128.58 to 1146.19)
	Anosmia	1.26 (0.58 to 2.84)	0.563	0.72 (0.51 to 1.02)	0.055	−1232.90 (−2983.05 to 517.26)

*Notes*: CI = confidence interval; NA = not applicable; OI = olfactory impairment; OR = odds ratio. ORs are from logistic regression models and cost ratios are exponentiated coefficients from gamma generalized linear models with a log-link function for healthcare/productivity cost, respectively. All models are adjusted for age, gender, ethnicity, body mass index, low socioeconomic status, smoking status, cardiovascular disease, diabetes, chronic kidney disease, asthma, hypertension, and dyslipidemia.

^a^Analyzed among subjects who are employed.

## Discussion

In our large, multiethnic, population-based study, we found that 34.0% of community-dwelling older adults ≥60 years had any OI, with 13.5% having anosmia. Factors that were linked to a higher likelihood of OI, particularly severe OI, included advanced age, male gender, regular alcohol consumption >4 times per week, and the presence of diabetes. In contrast, lower levels of moderate–vigorous physical activity and the presence of CKD were found to be protective. Our research also showed that OI, regardless of severity, was associated with a twofold increased risk of CI. These results should, however, be tempered by the discordance in OI prevalence rates when using within-population cut points to define OI. Considering these findings, further investigations incorporating objective electrophysiological OI measures to elicit Asian-specific cut points for OI should be carried out. These cut points can then be utilized in screening programs aimed at detecting and delaying the progression of OI to mitigate the potential impact of CI in older adults, which can have severe consequences for both patients and society.

Our census-weighted prevalence of 34.0% for the overall presence of any OI in community-dwelling older adults was substantially higher than the global average of objectively assessed OI (26.6%) reported in a meta-analysis by Desiato and colleagues in 2021 (8). Moreover, over a third of individuals with OI in our study were anosmic, emphasizing the need for screening and intervention programs to detect and delay OI progression in community-dwelling older adults. Compared with OI rates quantified via the SSIT in other Asian populations, our rates were higher than those of Taiwan (23.1%) (35), but lower than that of rural China (67.7%) (9). These results should, however, be interpreted with caution since the SSIT was developed and validated in a large population of Caucasian individuals (3,25). As such, its cut point for OI may not be applicable to Asia due to relative unfamiliarity with certain odors. Indeed, when we used the method pioneered by Hummel’s research group to derive population norms for the SSIT in our own population as detailed in the methods (25), we found a substantially reduced OI prevalence of 5.9%. These discrepant findings underscore the importance of culturally validating and establishing population norms for objective olfactory function assessments in Asia using functional electrophysiological tests, including OERPs and EOGs (5,6).

Our study found that older age and male gender were both associated with higher odds of OI across its severity spectrum, as previously reported (10,11). Several biological mechanisms have been proposed to underscore these associations, including age-related atrophy of the olfactory receptors and olfactory bulb, as well as the presence of higher levels of neuroprotective hormones like estrogen and progesterone in women (10,11). We also observed that having diabetes or more frequent alcohol consumption was associated with a higher likelihood of anosmia, which corroborates currently available data in the literature (9–11,36–38), although the underlying causative pathways remain unclear. Interestingly, our sensitivity analyses showed that compared with people of Chinese descent, individuals of Malay ethnicity had increased odds of having OI independent of other sociodemographic, medical, clinical, or lifestyle factors. This finding has not been reported previously, hence warranting further validation in other large-scale multiethnic Asian cohorts. Overall, our data suggest that targeted screening programs for these high-risk individuals, rather than community-wide screening efforts, may be a more practicable and cost-effective approach to address the growing problem of OI in older Asian adults. This approach may be particularly feasible in countries where community-wide screening programs for sensory impairments are already in place (39). For example, Singapore’s Ministry of Health has implemented Project Silver Screen, an annual screening program to detect vision and hearing impairment in older adults aged ≥60 years. Incorporating olfactory assessments within the sensory evaluation framework would not take a significant additional effort in this instance.

On the other hand, we found that both low moderate–vigorous levels of physical activity and the presence of CKD were associated with decreased odds of hyposmia. These findings are unexpected as other research groups have found the converse to be true, with hypothesized underpinning mechanisms supporting these outside findings (37,40–42). Our contradictory results could have arisen due to selection bias, that is, individuals in poorer health are less likely to have participated in the study. Recall and demand bias may also have played a contributory role as the duration of moderate–vigorous physical activity levels was based on self-report. Sensitivity analyses further revealed that a higher FMI was associated with lower likelihood of OI. This finding may be indicative of the obesity paradox, a phenomenon in which excess body fat serves as an alternative energy source, promoting enhanced functional health (43,44). Further longitudinal cohort studies are, therefore, warranted to validate these contrary findings.

We found that the presence of hyposmia and anosmia were associated with an over twofold increased odds of CI. Indeed, the potential of OI as a biomarker for CI is gaining recognition among researchers (45), as neurodegenerative changes characteristic of Alzheimer’s disease has been found in the central olfactory pathways in persons with mild CI; and the combination of OI assessments with neuroimaging measures has been found to predict the conversion rate to Alzheimer’s from MCI with a high degree of accuracy (46). These findings support the use of OI as a potential CI biomarker and underscores the need for further investigation on its potential contributory role in the pathogenesis of CI. Our findings should be interpreted with caution, however, as the 6-CIT and MoCA-Basic are not diagnostic tools for CI. Further investigations utilizing standard clinical diagnostic protocols, including neuropsychological test batteries and a neurologist assessment, are needed to substantiate our findings.

In contrast, no associations with other health indicators, including HRQoL, daily caloric intake, depressive symptoms, and frailty; or economic outcomes, that is, productivity loss and direct healthcare expenditure, were found. These results differ from several previously published studies showing a reduction in QoL (15); an increased risk of depression (14) and frailty (18); changes in caloric intake (16,17); and overall productivity loss (47), in individuals with OI. Several possible explanations could be put forward for the non-significant outcomes observed, including the use of a generic assessment tool like the EQ-5D, which may not have been sensitive enough to capture QoL challenges specific to OI (48). Differences in study population; as well as methodological disparities (eg, differences in how OI was assessed and defined), may also be a contributory factor to these discrepancies. For instance, the systematic review published by Kohli and colleagues on the relationship between OI and depression primarily included clinical case–control studies (14). As a result, the findings might have been influenced by selection bias and may not be generalizable to the general population. Additionally, recall and selection biases may have arisen from patients’ self-reporting of outcomes (eg, physical activity and exhaustion levels, both of which are essential components of the Fried frailty classification utilized in this analysis) and their inability to participate in the study due to poor health, respectively.

Strengths of this study include a large, well-characterized, and geographically representative study sample; our use of objective olfactory assessment to detect the presence of OI; and comprehensive multivariable adjustments for a range of relevant confounders. Limitations include the use of self-report to quantify certain risk determinants and outcome measures as mentioned previously, due to Singapore’s strict privacy laws that restrict access to participants’ electronic medical records, leading to the possibility of recall bias. This could be a key reason why we did not find any significant associations of known risk determinants of OI in our study, such as asthma, smoking, and the use of antidepressants, for example, cymbalta (10,11,49). In addition, we did not specifically record a history of comorbid upper respiratory trait disorders, for example, chronic rhinosinusitis, nasal polyposis, or previous nasal surgery; nor a previous history of COVID-19 infection as potential risk determinants of OI. In an attempt to resolve the latter issue, we undertook a comparison of the prevalence of OI between the years preceding the COVID-19 pandemic and the postpandemic years, which did not reveal any significant differences in the rates of OI, hyposmia, or anosmia (Supplementary Table 6), suggesting that the impact of COVID-19 on our population was minimal in this respect. Moreover, we did not conduct any cultural adaptation of the SSIT for our older Asian population. Although we carried out sensitivity analyses excluding odors that had poor identification rates across the whole population, the prevalence findings were particularly discordant, suggesting that our results may need to be interpreted with caution. In addition, we excluded individuals with dementia, severe deafness, and muteness from our study due to their inability to give informed consent; hence our results are not generalizable to these population subgroups. Lastly, our data are cross-sectional, and hence cause–effect inferences cannot be made. To address this gap, we are conducting a 4-year recall of PIONEER participants (PIONEER-2), which will enable us to better understand the risk factors and impact of hyposmia and anosmia over time both clinically and from the patient’s perspective.

In conclusion, we found that over one third of our population of community-dwelling older Asian adults aged ≥60 years had OI, with one in ten experiencing severe OI (anosmia). Given such a high prevalence rate and the substantially increased likelihood of developing CI in those with OI, it is imperative that targeted screening programs to detect and manage OI in high-risk individuals, particularly older men of Malay ethnicity, those with diabetes and frequent alcohol drinkers, be implemented to prevent or delay the development and progression of CI in these at-risk individuals.

## Supplementary Material

igae088_suppl_Supplementary_Table_S1-S6

## Data Availability

Study data can be made available upon reasonable request to the corresponding author. The study was not preregistered.
