# Supplementary material for: Understanding the Prevalence and Risk Factor Profile of Olfactory Impairment and Its Impact on Patient Health Indicators and Economic Outcomes in Community-Dwelling Older Asian Adults
Source: Innov Aging. 2024 Sep 27;8(10):igae088. doi: 10.1093/geroni/igae088 (PMC11472165; doi:10.1093/geroni/igae088)
Supplement: igae088_suppl_Supplementary_Table_S1-S6 [file igae088_suppl_supplementary_table_s1-s6.docx]

***Innovation in Aging* Supplementary Material: Man et al. Understanding the Prevalence and Risk Factor Profile of Olfactory Impairment; and its Impact on Patient Health Indicators and Economic Outcomes in Community-Dwelling Older Asian Adults.**

**Study Sampling Strategy**

In brief, 6,377 individuals were selected using an age-, gender-, and ethnicity- stratified sampling framework from a national database. Malays, Indians, females, and older age groups were deliberately oversampled to ensure adequate precision of estimates during statistical analyses. Study invitation letters were then sent out in batches, sorted by residential addresses; followed by home visits from study recruitment officers to ascertain an individual’s eligibility and agreement to participate. Of the invited individuals, 1,015 (15.9%) were classified as ‘uncontactable’ because of invalid address(s); were unresponsive to ≥3 home visit attempts and/or our recruitment officers were unable to access their residence(s) because of security restrictions. In addition, 648 (10.2%) individuals were excluded because they were incarcerated; were residing in nursing homes/outside Singapore; or were deceased; while a further 994 (15.6%) were deemed ineligible because they were terminally ill, bedridden or otherwise unable to give informed consent due to severe cognitive or hearing impairment or muteness. Of the remaining 3,720 (69.4%) eligible individuals, 2,643 (71.1%) took part in the study, 1,054 (28.3%) refused, and 22 (0.6%) were undecided (71.5% response rate). Reasons for refusal included lack of interest (n=895, 84.9%) or time needed to participate in the study (n=159, 15.1%). Compared to participants (n=2,643), non-participants (n=1,054) were older (p<0.001), more likely to be female (p<0.001), and more likely to be Chinese (p<0.001; all data not shown).

| **Supplementary Table 1.** Summary of the 16-item Sniffin' sticks test (identification component) | | | | |
| --- | --- | --- | --- | --- |
| **Item** | **No OI (N = 1260)** | **Hyposmia (N = 483)** | **Anosmia (N = 358)** | **Overall (N = 2101)** |
|  | **Mean (SD) or n (%)** | | | |
| Overall score | 12.5 (1.2) | 9.6 (0.5) | 6.5 (1.5) | 10.8 (2.6) |
| Median | 12 | 10 | 7 | 11 |
| 10th percentile | 11 | 9 | 4 | 7 |
| Pen 1: Orange |  |  |  |  |
| Orange | 1181 (93.7) | 393 (81.4) | 211 (58.9) | 1785 (85.0) |
| Blackberry | 11 (0.9) | 17 (3.5) | 20 (5.6) | 48 (2.3) |
| Strawberry | 26 (2.1) | 33 (6.8) | 65 (18.2) | 124 (5.9) |
| Pineapple | 37 (2.9) | 40 (8.3) | 60 (16.8) | 137 (6.5) |
| Pen 2: Leather |  |  |  |  |
| Leather | 865 (68.7) | 208 (43.1) | 80 (22.3) | 1153 (54.9) |
| Glue | 124 (9.8) | 91 (18.8) | 78 (21.8) | 293 (13.9) |
| Grass | 146 (11.6) | 111 (23.0) | 123 (34.4) | 380 (18.1) |
| Smoke | 107 (8.5) | 71 (14.7) | 72 (20.1) | 250 (11.9) |
| Pen 3: Cinnamon |  |  |  |  |
| Cinnamon | 1067 (84.7) | 328 (67.9) | 169 (47.2) | 1564 (74.4) |
| Chocolate | 32 (2.5) | 28 (5.8) | 35 (9.8) | 95 (4.5) |
| Honey | 46 (3.7) | 41 (8.5) | 52 (14.5) | 139 (6.6) |
| Vanilla | 110 (8.7) | 84 (17.4) | 98 (27.4) | 292 (13.9) |
| Pen 4: Peppermint |  |  |  |  |
| Peppermint | 1177 (93.4) | 378 (78.3) | 181 (50.6) | 1736 (82.6) |
| Chive | 37 (2.9) | 44 (9.1) | 74 (20.7) | 155 (7.4) |
| Fir | 21 (1.7) | 22 (4.6) | 38 (10.6) | 81 (3.9) |
| Onion | 21 (1.7) | 39 (8.1) | 63 (17.6) | 123 (5.9) |
| Pen 5: Banana |  |  |  |  |
| Banana | 1127 (89.4) | 339 (70.2) | 173 (48.3) | 1639 (78.0) |
| Coconut | 13 (1.0) | 23 (4.8) | 36 (10.1) | 72 (3.4) |
| Walnut | 49 (3.9) | 54 (11.2) | 63 (17.6) | 166 (7.9) |
| Cherry | 71 (5.6) | 67 (13.9) | 86 (24.0) | 224 (10.7) |
| Pen 6: Lemon |  |  |  |  |
| Lemon | 804 (63.8) | 221 (45.8) | 111 (31.0) | 1136 (54.1) |
| Peach | 158 (12.5) | 109 (22.6) | 116 (32.4) | 383 (18.2) |
| Apple | 52 (4.1) | 44 (9.1) | 37 (10.3) | 133 (6.3) |
| Grapefruit | 242 (19.2) | 108 (22.4) | 91 (25.4) | 441 (21.0) |
| Pen 7: Liquorice |  |  |  |  |
| Liquorice | 831 (66.0) | 200 (41.4) | 110 (30.7) | 1141 (54.3) |
| Cherry | 41 (3.3) | 39 (8.1) | 62 (17.3) | 142 (6.8) |
| Spearmint | 327 (26.0) | 195 (40.4) | 116 (32.4) | 638 (30.4) |
| Cookies | 50 (4.0) | 48 (9.9) | 65 (18.2) | 163 (7.8) |
| Pen 8: Turpentine |  |  |  |  |
| Turpentine | 672 (53.3) | 157 (32.5) | 85 (23.7) | 914 (43.5) |
| Mustard | 91 (7.2) | 62 (12.8) | 67 (18.7) | 220 (10.5) |
| Rubber | 176 (14.0) | 104 (21.5) | 93 (26.0) | 373 (17.8) |
| Menthol | 309 (24.5) | 159 (32.9) | 110 (30.7) | 578 (27.5) |
| Pen 9: Garlic |  |  |  |  |
| Garlic | 1169 (92.8) | 359 (74.3) | 183 (51.1) | 1711 (81.4) |
| Onion | 57 (4.5) | 74 (15.3) | 95 (26.5) | 226 (10.8) |
| Sauerkraut | 34 (2.7) | 45 (9.3) | 65 (18.2) | 144 (6.9) |
| Carrot | 0 (0.0) | 5 (1.0) | 15 (4.2) | 20 (1.0) |
| Pen 10: Coffee |  |  |  |  |
| Coffee | 1208 (95.9) | 410 (84.9) | 216 (60.3) | 1834 (87.3) |
| Cigarette | 22 (1.7) | 33 (6.8) | 52 (14.5) | 107 (5.1) |
| Wine | 16 (1.3) | 9 (1.9) | 33 (9.2) | 58 (2.8) |
| Smoke | 14 (1.1) | 31 (6.4) | 57 (15.9) | 102 (4.9) |
| Pen 11: Apple |  |  |  |  |
| Apple | 274 (21.7) | 64 (13.3) | 35 (9.8) | 373 (17.8) |
| Melon | 192 (15.2) | 115 (23.8) | 109 (30.4) | 416 (19.8) |
| Peach | 738 (58.6) | 262 (54.2) | 155 (43.3) | 1155 (55.0) |
| Orange | 53 (4.2) | 42 (8.7) | 59 (16.5) | 154 (7.3) |
| Pen 12: Clove |  |  |  |  |
| Clove | 1048 (83.2) | 281 (58.2) | 136 (38.0) | 1465 (69.7) |
| Pepper | 38 (3.0) | 38 (7.9) | 63 (17.6) | 139 (6.6) |
| Cinnamon | 135 (10.7) | 130 (26.9) | 115 (32.1) | 380 (18.1) |
| Mustard | 37 (2.9) | 34 (7.0) | 44 (12.3) | 115 (5.5) |
| Pen 13: Pineapple |  |  |  |  |
| Pineapple | 1020 (81.0) | 292 (60.5) | 133 (37.2) | 1445 (68.8) |
| Pear | 63 (5.0) | 43 (8.9) | 62 (17.3) | 168 (8.0) |
| Plum | 90 (7.1) | 64 (13.3) | 77 (21.5) | 231 (11.0) |
| Peach | 85 (6.7) | 84 (17.4) | 86 (24.0) | 255 (12.1) |
| Pen 14: Rose |  |  |  |  |
| Rose | 936 (74.3) | 221 (45.8) | 122 (34.1) | 1279 (60.9) |
| Camomile | 194 (15.4) | 143 (29.6) | 86 (24.0) | 423 (20.1) |
| Raspberry | 78 (6.2) | 68 (14.1) | 78 (21.8) | 224 (10.7) |
| Cherry | 48 (3.8) | 50 (10.4) | 71 (19.8) | 169 (8.0) |
| Pen 15: Anise |  |  |  |  |
| Anise | 1167 (92.6) | 366 (75.8) | 164 (45.8) | 1697 (80.8) |
| Rum | 32 (2.5) | 44 (9.1) | 54 (15.1) | 130 (6.2) |
| Honey | 30 (2.4) | 51 (10.6) | 90 (25.1) | 171 (8.1) |
| Fir | 29 (2.3) | 22 (4.6) | 45 (12.6) | 96 (4.6) |
| Pen 16: Fish |  |  |  |  |
| Fish | 1203 (95.5) | 410 (84.9) | 229 (64.0) | 1842 (87.7) |
| Bread | 9 (0.7) | 13 (2.7) | 20 (5.6) | 42 (2.0) |
| Cheese | 18 (1.4) | 16 (3.3) | 39 (10.9) | 73 (3.5) |
| Ham | 29 (2.3) | 44 (9.1) | 67 (18.7) | 140 (6.7) |

Notes. OI: Olfactory Impairment; SD: Standard Deviation.

| **Supplementary Table 2.** Sensitivity analysis **-** Prevalence of any OI,hyposmia and anosmia, stratified by age, gender and ethnicity in the PIONEER Study | | | | | | | | | | | | | |
| --- | --- | --- | --- | --- | --- | --- | --- | --- | --- | --- | --- | --- | --- |
|  | **All (*N* = 2101)** | | | **Gender** | | | | **Ethnicity** | | | | | |
|  |  |  |  | **Male (*n* = 944)** | | **Female (*n* = 1157)** | | **Chinese (*n* = 1010)** | | **Malay (*n* = 605)** | | **Indian (*n* = 486)** | |
|  | ***n*** | | **Weighted, %  (95% CI)** | ***n*** | **Weighted, %  (95% CI)** | ***n*** | **Weighted, %  (95% CI)** | ***n*** | **Weighted, %  (95% CI)** | ***n*** | **Weighted, %  (95% CI)** | ***n*** | **Weighted, %  (95% CI)** |
| **Any OI (hyposmia)** | | | | | | | | | | | | | |
| 60-69 | | 24 | 2.7 (1.5, 4.4) | 13 | 2.2 (0.9, 4.5) | 11 | 3.1 (1.3, 6.1) | 9 | 2.5 (1.1, 4.7) | 8 | 3.0 (1.3, 5.9) | 7 | 3.8 (1.6, 7.7) |
| 70-79 | | 50 | 6.8 (4.8, 9.5) | 28 | 7.3 (4.2, 11.6) | 22 | 6.5 (3.9, 10.0) | 25 | 6.7 (4.4, 9.8) | 17 | 8.8 (5.2, 13.7) | 8 | 5.4 (2.4, 10.4) |
| ≥ 80 | | 100 | 17.3 (13.3, 22.0) | 52 | 18.2 (12.6, 25.0) | 48 | 16.8 (11.4, 23.4) | 47 | 16.8 (12.3, 22.2) | 26 | 20.4 (13.4, 28.9) | 27 | 20.7 (14.1, 28.7) |
| *p*-trend | | <0.001 | | <0.001 | | <0.001 | | <0.001 | | <0.001 | | <0.001 | |
| Total | | 174 | 5.9 (4.8, 7.2) | 93 | 5.5 (4.0, 7.3) | 81 | 6.3 (4.6, 8.2) | 81 | 5.8 (4.5, 7.4) | 51 | 6.2 (4.5, 8.3) | 42 | 6.3 (4.3, 8.8) |
| Notes. CI: Confidence Interval; OI: Olfactory Impairment. Weighted prevalences were calculated with sampling weights specific to each age group, gender and ethnicity to adjust for oversampling and post-stratification weights to align to the population distribution based on the 2020 Singapore Census. Any OI (hyposmia) is defined using score of < 7 (out of 14). | | | | | | | | | | | | | |

| **Supplementary Table 3.** Sensitivity analysis - Risk determinants of any OI across its severity spectrum (hyposmia, anosmia) | | |
| --- | --- | --- |
| **Variable** | **OR (95% CI)** | **P-value** |
| Age (year) | 1.12 (1.08, 1.17) | <0.001 |
| Gender |  |  |
| Female | Reference | NA |
| Male | 0.86 (0.41, 1.78) | 0.695 |
| Ethnicity |  |  |
| Chinese | Reference | NA |
| Malay | 2.38 (1.13, 5.14) | 0.024 |
| Indian | 1.48 (0.63, 3.40) | 0.355 |
| High FMI |  |  |
| No | Reference | NA |
| Yes | 0.50 (0.26, 0.92) | 0.028 |
| Low socioeconomic status |  |  |
| No | Reference | NA |
| Yes | 0.75 (0.33, 1.55) | 0.455 |
| Smoking status |  |  |
| Never smoked | Reference | NA |
| Past smoker | 2.08 (0.97, 4.53) | 0.061 |
| Current smoker | 1.87 (0.65, 5.09) | 0.230 |
| Alcohol consumption |  |  |
| None | Reference | NA |
| ≤ 4 days/week | 1.69 (0.65, 4.06) | 0.259 |
| > 4 days/week | 2.76 (0.69, 8.81) | 0.109 |
| Low MVPA level |  |  |
| No | Reference | NA |
| Yes | 1.17 (0.65, 2.15) | 0.598 |
| CVD |  |  |
| No | Reference | NA |
| Yes | 1.50 (0.75, 2.90) | 0.242 |
| Diabetes |  |  |
| No | Reference | NA |
| Yes | 1.04 (0.56, 1.88) | 0.904 |
| CKD |  |  |
| No | Reference | NA |
| Yes | 0.91 (0.45, 1.75) | 0.774 |
| Asthma |  |  |
| No | Reference | NA |
| Yes | 0.88 (0.28, 2.24) | 0.808 |
| Hypertension |  |  |
| No | Reference | NA |
| Yes | 0.56 (0.25, 1.34) | 0.170 |
| Dyslipidemia |  |  |
| No | Reference | NA |
| Yes | 1.47 (0.55, 5.13) | 0.491 |
| Neurological disorders |  |  |
| No | Reference | NA |
| Yes | 0.60 (0.17, 1.61) | 0.361 |
| Neuropsychiatric disorders |  |  |
| No | Reference | NA |
| Yes | 1.15 (0.25, 3.80) | 0.836 |
| Notes. BP: Blood Pressure; CI: Confidence Interval; MVPA: Moderate-Vigorous Physical Activity; OI: Olfactory Impairment; OR: Odds Ratio. High FMI: High fat mass index (FMI) defined as FMI > 8.51 for males and FMI > 11.6 for females; Low Socioeconomic Status: Having primary or lower education and individual monthly income < SGD2000; Low MVPA level: Gender-specific lowest quintile of total self-reported duration spent carrying out moderate and vigorous activity (e.g., gardening, brisk walking, dancing, jogging); Cardiovascular Disease (CVD): Self-reported history of angina, heart attack, heart disease or stroke; Diabetes: HbA1c > 6.5%, random blood glucose ≥ 11.1 mmol/L, use of diabetic medication and/or self-reported; Chronic Kidney Disease (CKD): Estimated glomerular filtration rate <60ml/min/1.73m2; Asthma: Self-reported; Hypertension: Systolic BP ≥ 140mmHg, diastolic BP ≥ 90mmHg, physician diagnosis, use of BP medication and/or self-report; Dyslipidemia: Total cholesterol ≥ 5.2 mmol/L or LDL cholesterol ≥ 3.4 mmol/L or triglycerides ≥ 1.7 mmol/L or use of anti-cholesterol medication; Neurological disorders: Self-reported history of neurological disorders (e.g., Parkinsons, multiple sclerosis, migraines); Neuropsychiatric disorders: Self-reported history of neuropsychiatric (including depression) disorders or any use of duloxetine (Cymbalta) or the class of selective serotonin reuptake inhibitors (SSRIs) medications. | | |

| **Supplementary Table 4.** Sensitivity analysis - Multivariable associations between any OI and key health indicators | | | | | |
| --- | --- | --- | --- | --- | --- |
| **Health Indicators** | **Exposure** | **Estimate** ^a^ **(95% CI)** | ***p*-value** | **Overall Marginal Effect (95% CI)** | **% change** |
| EQ-5D (HRQoL) | No OI | Reference | NA |  |  |
|  | Any OI (Hyposmia) | -0.013 (-0.037, 0.012) | 0.314 | -0.013 (-0.037, 0.012) | -1.39 |
| PHQ-9 score (depressive symptoms) | No OI | Reference | NA |  |  |
|  | Any OI (Hyposmia) | -0.059 (-0.449, 0.332) | 0.768 | -0.059 (-0.449, 0.331) | -6.91 |
| Daily caloric intake (kcal/day) | No OI | Reference | NA |  |  |
|  | Any OI (Hyposmia) | 11.46 (-133.76, 156.68) | 0.877 | 11.46 (-133.61, 156.53) | 0.61 |
| Frailty | No OI | Reference | NA |  |  |
|  | Any OI (Hyposmia) | OR: 0.84 (0.43, 1.54) | 0.578 | -0.018 (-0.077, 0.042) | -13.91 |
| Cognitive impairment | No OI | Reference | NA |  |  |
|  | Any OI (Hyposmia) | OR: 1.84 (0.99, 3.35) | 0.049 | 0.049 (-0.007, 0.104) | 74.75 |

Notes. BMI: Body Mass Index; CI: Confidence Interval; EQ-5D: EuroQoL 5-dimension; Health-Related Quality of Life; OI: Olfactory Impairment; OR: Odds Ratio; PHQ-9: Patient Health Questionnaire-9; PRO: Patient-Reported Outcome. All models are adjusted for age, gender, ethnicity, BMI, low socioeconomic status, smoking status, cardiovascular disease, diabetes, chronic kidney disease, asthma, hypertension and dyslipidemia.
^a^ For PROs EQ-5D, PHQ-9 and daily caloric intake, the estimates are coefficients derived from linear regression models. The remaining PROs frailty and cognitive impairment are odds ratios (OR) derived from logistic regression models.

| **Supplementary Table 5**. Associations between OI (hyposmia) and economic outcomes using two-parts model | | | | | | |
| --- | --- | --- | --- | --- | --- | --- |
| **Cost outcome** | **Exposure** | First part | | Second part | | **Overall Marginal Effect (95% CI)** |
|  |  | **OR**  **(95% CI)** | ***p*-value** | **Cost Ratio**  **(95% CI)** | ***p*-value** |  |
| Healthcare cost |  |  |  |  |  |  |
|  | No OI | Reference | NA | Reference | NA |  |
|  | Hyposmia | 0.54 (0.30, 0.93) | 0.03 | 0.89 (0.30, 3.64) | 0.839 | -152.00 (-421.47, 117.47) |
|  |  |  |  |  |  |  |
| Productivity cost ^a^ |  |  |  |  |  |  |
|  | No OI | Reference | NA | Reference | NA |  |
|  | Hyposmia | 1.34 (0.36, 5.68) | 0.671 | 0.67 (0.39, 1.26) | 0.176 | -1371.45 (-3994.26, 1251.35) |

Notes. CI: Confidence Interval; OI: Olfactory Impairment; OR: Odds Ratio. ORs are from logistic regression models and cost ratios are expoentiated coefficients from gamma generalised linear models with a log-link function for healthcare/productivity cost respectively. All models are adjusted for age, gender, ethnicity, body mass index, low socioeconomic status, smoking status, cardiovascular disease, diabetes, chronic kidney disease, asthma, hypertension and dyslipidemia.
^a^ Analysed among subjects who are employed.

| **Supplementary Table 6.** Comparison of the census-weighted prevalence of OI, hyposmia and anosmia pre- and post-COVID-19 | | | | |
| --- | --- | --- | --- | --- |
|  | **Pre-COVID-19 (N = 981)** | | **Post-COVID-19 (N = 1120)** | |
|  |  |  |  |  |
|  | **n** | **Weighted, %  (95% CI)** | **n** | **Weighted, %  (95% CI)** |
| **Any OI** | | |  | |
| 60-69 | 82 | 24.3 (19.1, 30.1) | 111 | 24.7 (19.9, 30.1) |
| 70-79 | 141 | 40.4 (34.3, 46.8) | 161 | 42.1 (35.7, 48.6) |
| ≥ 80 | 151 | 50.3 (42.6, 58.1) | 195 | 68.9 (60.3, 76.6) |
|  | P-trend <0.001 | | P-trend <0.001 | |
| Total | 374 | 33.0 (29.4, 36.8) | 467 | 35.1 (31.5, 38.8) |
| **Hyposmia** | | |  | |
| 60-69 | 57 | 16.6 (12.2, 21.8) | 70 | 15.8 (11.8, 20.6) |
| 70-79 | 81 | 23.1 (18.0, 28.8) | 104 | 26.8 (21.3, 33.0) |
| ≥ 80 | 79 | 28.3 (21.6, 35.9) | 92 | 30.3 (23.0, 38.3) |
|  | P-trend = 0.001 | | P-trend <0.001 | |
| Total | 217 | 20.3 (17.2, 23.6) | 266 | 20.7 (17.7, 24.1) |
| **Anosmia** | | |  |  |
| 60-69 | 25 | 7.7 (4.7, 11.8) | 41 | 8.9 (5.8, 12.9) |
| 70-79 | 60 | 17.4 (12.9, 22.7) | 57 | 15.2 (10.9, 20.5) |
| ≥ 80 | 72 | 22.0 (16.3, 28.7) | 103 | 38.6 (30.4, 47.4) |
|  | P-trend <0.001 | | P-trend <0.001 | |
| Total | 157 | 12.7 (10.4, 15.4) | 201 | 14.4 (11.9, 17.2) |
| Notes. CI: Confidence Interval; OI: Olfactory Impairment. Weighted prevalences were calculated with sampling weights specific to each age group, gender and ethnicity to adjust for oversampling and post-stratification weights to align to the population distribution based on the 2020 Singapore Census. Pre-COVID period is before 7th April 2020. | | | | |
